# Silent Fistula of the Ascending Aorta to Pericardium by Brucella Endocarditis

**DOI:** 10.15171/jcvtr.2015.28

**Published:** 2015

**Authors:** Feridoun Sabzi, Siavoosh Vaziri, Reza Faraji

**Affiliations:** ^1^ Preventive Cardiovascular Research Center Kermanshah, Kermanshah University of Medical Sciences, Kermanshah, Iran; ^2^ Department of Infectious Diseases and Tropical Medicine, Imam Reza Hospital, Kermanshah University of Medical Sciences, Kermanshah, Iran; ^3^ Yazd Cardiovascular Research Center, Shahid Sadoughi University of Medical Sciences, Yazd, Iran

**Keywords:** Endocarditis, Brucella, Heart

## Abstract

We report the case of a 26-year-old male patient with 2-week history of Brucella aortic valve endocarditis that was referred from general hospital to our hospital emergency room with pallor of the skin and mucous membranes accompanied by systemic hypotension and chest pain. Trans esophageal echocardiography (TEE) revealed a 30-mm ascending aorta at the pulmonary trunk with no evidence of the false lumen or intimal flap. TEE also showed a large vegetation of the aortic valve that limited to noncoronary sinus with moderate pericardial effusion. TEE did not showed fistula tract of nonaortic coronary sinus ring to intra mural of aorta and to pericardial cavity. The patient underwent open heart surgery with resection of destructed aortic valve and vegetation and replacement of aortic valve with prosthetic valve (Carbomedics, Sorin group. 23 mm sizes) with separated pledged suture. Debridement of aortic intra mural fistula tract and its replacement with fresh pericardial patch than performed. The pericardial cavity had moderate bloody effusion. The patient recovered uneventfully and was discharged in the 15th postoperative day. In this case, we report a rare silent clinical presentation of aortic wall fistula by vegetation and aortic ring abscess and periaortic wall hematoma, and reviewed its medical and surgical treatment.

## Introduction

Brucella endocarditis is an uncommon infection of cardiovascular system and its incidence among the rare and fastidious bacteria as a causative agent in endocarditis is estimated to be 5%. In a review article of endocarditis caused by unusual bacteria in recorded 120 cases of endocarditis only, 6 cases were related to Brucella endocarditis.^1^ Brucella infection usually affected aortic root apparatus such as aortic valve, aortic ring and fistula formation to surrounding tissue. The aortic valve lesions were presented as voluminous and ulcerative vegetation, associated with large abscesses cavity penetrated to neighboring tissue as the myocardium, microabscesses formation over the aortic cusps, rupture of the commissures, and calcification of valve and ring.^2^ Involvement of normal aortic tissue by Brucella infection is very rare event. Careful literature review showed that aortic wall with some different pathology such as presence of atheroma and aneurysm is predisposed to involvement by Brucella infection. In very rare cases aneurismal wall of aorta infected by Brucella agent during septicemia. Passage of bacteria to aortic wall occurred via vasa vasorum that named mycotic aneurysm. Retrograde ascending aorta involvement by aortic root Brucella is exceedingly rare event that here we report it as intra mural fistula originate from ring and extending upward as a fistula to pericardium.^3^ The natural history of this type of fistula is unknown it may progress to rupture in pericardial cavity or lead to dissection of ascending aorta, or may be regressed with suitable medical therapy of aortic valve endocarditis. It seems that this type of fistula is a step in the progression of aortic valve endocarditis. Ferrero showed that Brucella has specific tendency to penetrate endothelial cells of vascular system and with infection of endothelial cell caused a strong inflammatory reaction, leading to predicted cardiovascular sequels.^4^ However endothelial cells of native valves is the most common site of Brucella invasion in cardiovascular system but vascular involvement, may be includes any arterial vessels as ascending aorta, subclavian artery, brachial artery, or cerebral arteries or any veins.^5^ Vascular involvement by Brucella may associated with or without underlying endocarditis. The most important factor in vascular complication of Brucella is endothelial cell infection and subsequent inflammatory reaction and the immune response to bacteria antigens in the vessel wall are considered as probable mechanisms for pathologic complication of vessel wall as aortic involvement. Aortic endothelial activation in reaction to Brucella invasion is associated with up regulation of adhesion of Brucella antigen–antibody complex followed by release of proinflammatory cytokine and chemokine. These complex inflammatory reactions may play an important role in aortic wall injury and fistula formation to surrounding tissues as pericardial cavity.^6^ Management of this condition varies according to its location and extension of aortic wall destruction; however there is no consensus to early surgical indication for patients with Brucella aortic valve endocarditis in whom no complicating factors such as hemodynamic instability, pain, rupture, imminent rupture or signs of cardiac tamponade was found.^1-3^ This article reports the case of a patient with a complicated intramural fistula tract of aortic ring to aorta wall who underwent a successful surgical treatment.

## Case Report

**Figure 1:**
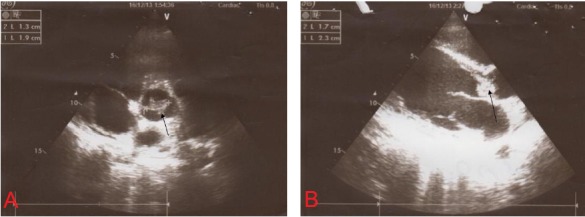
(A) Shows large vegetation on aortic valve. (B) Shows vegetation and thickness of aortic wall.

**Figure 2:**
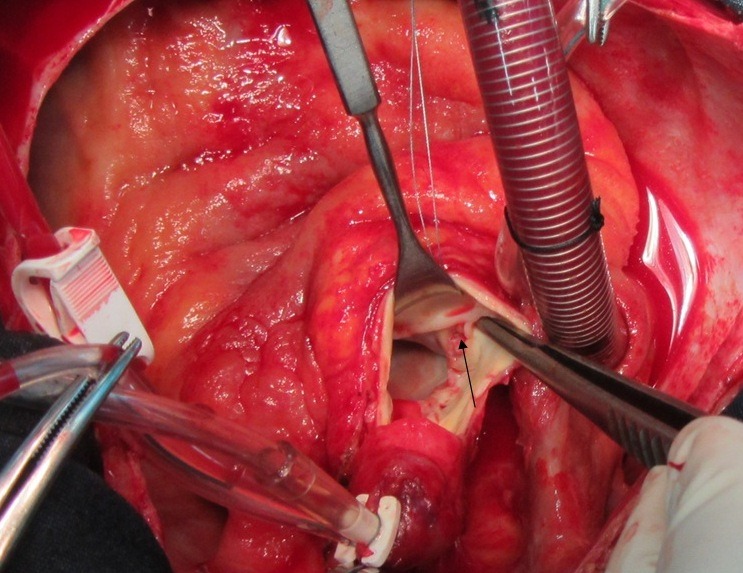
Shows site of ring perforation in non-coronary sinus after resection of vegetation and aortic valve (black arrow).

**Figure 3:**
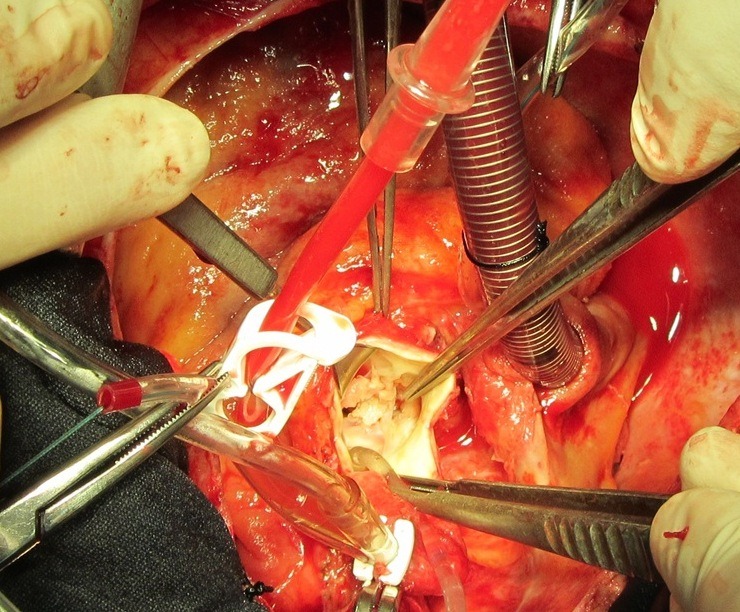
Shows large vegetation on aortic valve.

**Figure 4:**
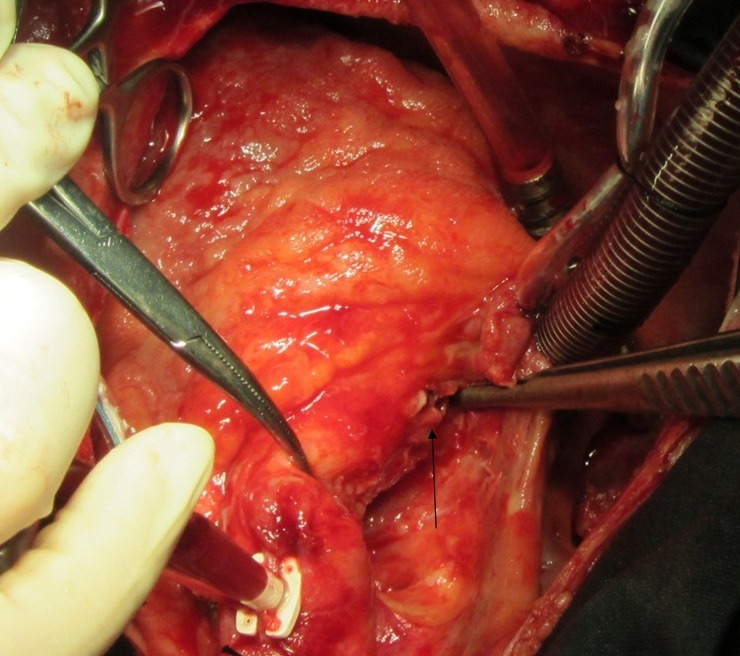
Shows inflammation of epicardial surface of heart and external crater of fistula after removal of perifistula hematoma.

**Figure 5:**
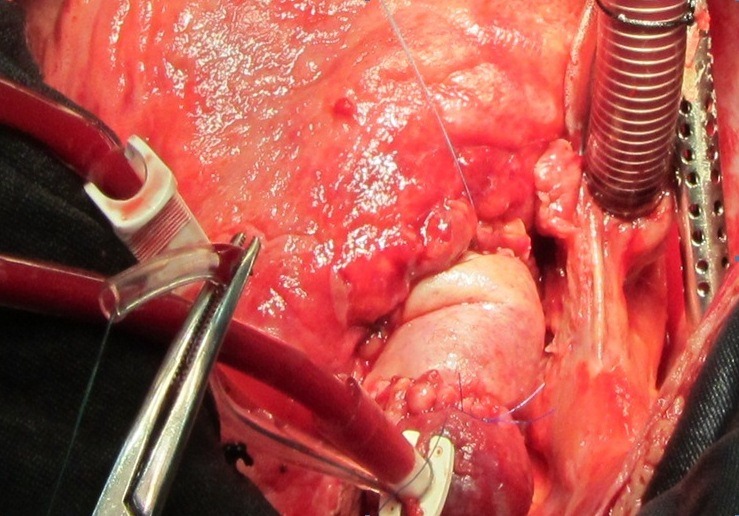
Shows repair of aortic wall by fresh pericardial patch.

Twenty-six-year-old male patient referred from general hospital and admitted to our center with a history of Brucella aortic valve endocarditis, accompanied by hypotension and chest pain. Chest radiography showed moderately enlarged cardiac silhouette and mediastinum. Results of laboratory tests made on admission were as follow: White blood cell count, 19000/mm^-3^ with 91% neutrophils; platelet count, 89000/mm^-3^ ; hemoglobin, 8 g/dl; c-reactive protein, 54 mg/dl; erythrocyte sedimentation rate, 80 mm/h; blood urea nitrogen (BUN), 40 mg/dL; creatine (Cr), 2.9 mg/dL; and urinalysis revealed no any hematuria and 24-hour urinalysis (U.A) revealed proteinuria). Antinuclear antibody (ANA), antineutrophil cytoplasmic antibody (ANCA ), anti-double stranded DNA, rheumatic factor (RF), anticardiolopin antibody, lupus anticoagulant were all negative. Serum agglutination tests were positive (titer>1:1500), and enzyme-linked immunosorbent assay tests for anti-Brucella IgG and IgM antibodies were strongly positive (133 U/ml and 31.7 U/ml; 30 U/ml and 20 U/ml, respectively). In addition, Brucella melitensis was isolated from 2 of 3 consecutive blood cultures. No significant alterations were observed in the electrocardiogram. TEE revealed normal aortic diameter at the mid-ascending aorta, severe aortic regurgitation, aortic ring abscess in noncoronary sinus, large vegetation in noncoronary sinus leaflet with preserved ventricular function and moderate pericardial effusion (Figure 1A and  1B). Aortic wall fistula from ring abscess to pericardium temporary was blocked by intra fistula vegetation. Intraoperative finding revealed large vegetation and perifistula hematoma in aortic wall. The fistula tract starting from aortic ring (Figures 2 and 3) and extended to aortic wall as intramural tract in a distance of 15 mm above the ring and ending to external aortic wall as a crater of fistula (Figure 4). We think that initial bleeding from fistula tract to pericardium was blocked by vegetation that confirmed intra operatively. The patient then underwent conventional surgical treatment with extracorporeal circulation (ECC), and replacement of aortic valve with prosthetic valve (carbomedic, sorin group, 23 mm size) and resection of aortic wall containing fistula site and its replacement with fresh pericardial patch (Figure 5). Patient treated with ceftriaxone 2 g and gentamicin 3 mg/kg prior to culture report. Therapy with doxycycline 100 mg twice daily with gentamicin 3 mg/kg intravenus in 2 divided doses with oral rifampin 600 mg once daily for 6 weeks was started after confirmation of Brucella endocarditis. The patient recovered uneventfully and was discharged in the 15th postoperative day.

## Discussion

Brucellosis endocarditis is a zoonosis infection of cardiovascular system with worldwide distribution, which is endemic in many provinces of the Iran. Brucella endocarditis is due to direct invasion of organism to the endocardium and neighboring organ as pericardium.^4^ Valvular involvements include valve cusp destruction, destruction of commissures, rupture of chordae, aortic valve ring myocardial abscess, and rupture to pericardium.^5^ Keshtkar-Jahromi et al^6^ showed that Brucella endocarditis is a destructive process with high tendency to tissue ulceration, leading to severe injury of valves, and large vegetations which are accompanied by tissue lyses and fistula formation.Jeroudi et al^7^ reported 4 adult patients with normal hearts who developed severe aortic regurgitation after infection with Brucella, and in one of them mitral valve was also affected. At operation all patients had aortic valve vegetation and combination of severe destruction of valves structures. Three patients had cusp perforation, and one aortic root abscess. The aortic valve was affected in most of cases, and the incidence of endocarditis associated with cardiac abscesses was 20.5%. Colmenero et al^8^ have described several reasons for low incidence of positive blood culture in Brucella endocarditis such as intracellular location and fastidious nature of organism, previous treatment with antibiotics, and long duration between beginnings of symptoms and the diagnosis.The intracellular location of the organism makes it unavailable for antibiotics. Our case is the first description of Brucella aortic valve endocarditis that complicated by silent fistula to pericardium and moderate pericardial effusion. The most common complication of cardiovascular system of this disease, is endocarditis, however myocarditis and pericarditis, are very rare, but aortic valve endocarditis is more fatal than involvement of others parts of heart. Native aortic valve endocarditis is a fatal complication of brucellosis, often more common than others native valves. Arnett et al^9^ described that onset of pericarditis with effusuion may indicate an annulus abscess rupturing into the pericardial space.The present report, describes an exceptional case of silent fistulization betweenthe non coronary sinus of aorta and pericardium by *Brucella melitensis* in a 26 years old patient. The patient present with fever and weight loss and arthralgia in first admission. He was strongly positive for brucellosis by serological reaction and conventional microbiological cultures form blood and valve tissue were successful. The patients renal dysfunction was related to effect of chronic inflammation (endocarditis) on renal function and prolonged nephrotoxic drug usage and anemia could be described by chronic inflammation that exaggerated by intrapericardial bleeding to pericardium by small fistula. Echocardiography revealed aortic root abscess, cavity formation in aortic ring, large vegetation and native aortic valve destruction with aortic regurgitation and fistula from non coronary sinus to pericardium and moderate bloody effusion. The intraoperative exploration of aortic valve revealed that this fistula was blocked by vegetation in fistula tract. After a short course of antibiotic therapy, the patient underwent open heart surgery with aortic valve replacement and trance pulmonary fistula repair. The patient had uneven full postoperative recovery and with good general condition discharged with oral antibiotics to home in 15th days of hospitalization.

## Conclusion

In this exceedingly case report we report silent clinical presentation of aortic wall fistula to pericardium caused by aortic ring abscess and destruction of aortic wall, and reviewed its medical and surgical treatment.

## Ethical Issues

The study was approval by our local Ethics Committee.

## Competing Interests

Authors declare no conflict of interest in this study.

## References

[1] Inan MB, Eyileten ZB, Ozcinar E (2010). Native valve Brucella endocarditis. Clin Cardiol.

[2] Al Dahouk S, Schneider T, Jansen A, Nöckler K, Tomaso H, Hagen RM (2006). Brucella endocarditis in prosthetic valves. Can J Cardiol.

[3] Solera J, Martínez-Alfaro E, Sáez L (1994). Meta-analysis of the efficacy of the combination of rifampicin and doxycycline in the treatment of human brucellosis. Med Clin (Barc).

[4] Ferrero MC, Bregante J, Delpino MV, Barrionuevo P, Fossati CA, Giambartolomei GH (2011). Proinflammatory response of human endothelial cells to Brucella infection. Microbes Infect.

[5] Lubani M, Sharda D, Helin I (1986). Cardiac manifestations in brucellosis. Arch Dis Child.

[6] Keshtkar-Jahromi M, Razavi SM, Gholamin S, Hossain M, Sajadi MM (2012). Medical versus medical and surgical treatment for Brucella endocarditis. Ann Thorac Surg.

[7] Jeroudi MO, Halim MA, Harder EJ, Al-Siba’i MB, Ziady G, Mercer EN (1987). Brucella endocarditis. Br Heart J.

[8] Colmenero JD, Reguera JM, Martos F, Sánchez-De-Mora D, Delgado M, Causse M (1996). Complications associated with Brucella melitensis infection: a study of 530 cases. Medicine (Baltimore).

[9] Arnett EN, Roberts WC (1976). Valve ring abscess in active infective endocarditis Frequency, location, and clues to clinical diagnosis from the study of 95 necropsy patients. Circulation.

